# A LiDAR-Camera Joint Calibration Algorithm Based on Deep Learning

**DOI:** 10.3390/s24186033

**Published:** 2024-09-18

**Authors:** Fujie Ren, Haibin Liu, Huanjie Wang

**Affiliations:** College of Mechanical and Energy Engineering, Beijing University of Technology, Beijing 100124, China; renfujie80@emails.bjut.edu.cn (F.R.); wanghuanjie@bjut.edu.cn (H.W.)

**Keywords:** deep learning, automatic driving, LiDAR-camera calibration, feature extraction

## Abstract

Multisensor (MS) data fusion is important for improving the stability of vehicle environmental perception systems. MS joint calibration is a prerequisite for the fusion of multimodality sensors. Traditional calibration methods based on calibration boards require the manual extraction of many features and manual registration, resulting in a cumbersome calibration process and significant errors. A joint calibration algorithm for a Light Laser Detection and Ranging (LiDAR) and camera is proposed based on deep learning without the need for other special calibration objects. A network model constructed based on deep learning can automatically capture object features in the environment and complete the calibration by matching and calculating object features. A mathematical model was constructed for joint LiDAR-camera calibration, and the process of sensor joint calibration was analyzed in detail. By constructing a deep-learning-based network model to determine the parameters of the rotation matrix and translation matrix, the relative spatial positions of the two sensors were determined to complete the joint calibration. The network model consists of three parts: a feature extraction module, a feature-matching module, and a feature aggregation module. The feature extraction module extracts the image features of color and depth images, the feature-matching module calculates the correlation between the two, and the feature aggregation module determines the calibration matrix parameters. The proposed algorithm was validated and tested on the KITTI-odometry dataset and compared with other advanced algorithms. The experimental results show that the average translation error of the calibration algorithm is 0.26 cm, and the average rotation error is 0.02°. The calibration error is lower than those of other advanced algorithms.

## 1. Introduction

**Figure 1:**
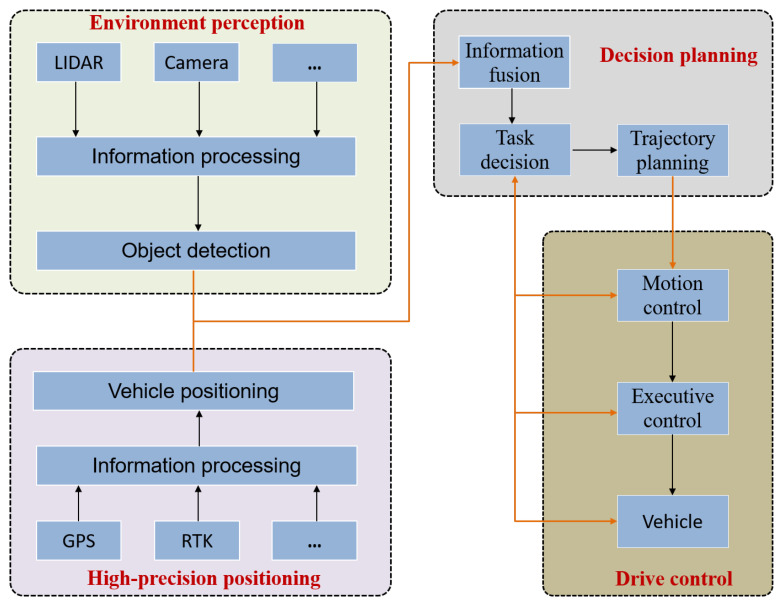
Automatic driving system module flow chart.

Recently, autonomous-driving technology has been developed for fully automated driving [1]. However, due to such limitations as traffic coordination requirements and difficulty in algorithm development, the most advanced level of intelligent autonomous driving has reached a high level of automation but still cannot achieve complete automation. Generally, an autonomous-driving system comprises four modules: environmental perception, high-precision positioning, decision planning, and drive control. A flow chart of the four system modules of the autodrive system is shown in Figure 1. The main task of an environmental perception system is to obtain spatial information about the surrounding environment and identify obstacle information using sensing devices such as LiDAR and cameras [2]. The main task of high-precision positioning systems is to determine the positions of autonomous vehicles in space using positioning systems such as the Global Positioning System (GPS). The decision-planning system is designed to provide different types of feedback and operations to vehicles based on known perception information and artificially set scenarios. The last part is the drive control system. When the decision-planning system provides operational instructions, various parts of the vehicle complete the corresponding operations according to the instructions. One obstacle hindering the development of autonomous driving technology is the need for autonomous vehicles to respond in real time to complex and ever-changing traffic environments. Therefore, how to improve the environmental perception ability of autonomous-driving vehicles is an urgent problem that needs to be solved at present.

As shown in Figure 1, the process of environmental perception mainly relies on sensors that check the surrounding environment, identify targets, and send perception results to the decision-planning module. The effectiveness of environmental perception depends on the selection of sensors, optimization of perception algorithms, and complexity of the surrounding environment. With the rapid development of autonomous-driving technology, an increasing number of cars are equipped with sensors, such as LiDAR and cameras, to perceive real-time environmental information while driving. Based on the perception results, tasks such as target recognition, dynamic obstacle avoidance, and path planning are completed to improve the intelligence of autonomous vehicles. Therefore, this paper will study an efficient joint calibration algorithm of LiDAR and camera to improve the environmental awareness of autonomous-driving vehicles.

An environmental-sensing system based on a single-source sensor has limitations, among which the environmental-sensing system based on a single camera is susceptible to changes in light intensity, and the system’s stability is poor. There is less texture information in the point cloud image collected by an environmental-sensing system based on a single LiDAR, and the detection accuracy is low. The effective integration of LiDAR and a camera [3,4,5] can provide more abundant and more accurate raw data for the environmental-perception system of autonomous vehicles and improve the stability and detection accuracy of the perception system. The joint calibration of sensors is the premise and basis of fusion [6,7,8].

Currently, calibration methods for LiDAR sensors and cameras can be divided into target-based and target-free methods. The target-based method utilizes specific calibration targets to extract and match features during the calibration process to obtain external parameters between the camera and the LiDAR sensor. This method requires the use of special calibration objects, such as chessboard calibration boards. During the calibration process, a large number of calibration board feature points must be manually extracted for matching. Geiger et al. [9] proposed a chessboard angle detector for camera and LiDAR calibration. Guo et al. [10] presented a solution to the 2D LiDAR camera extrinsic calibration problem Tekla et al. [6] proposed an external calibration method for a laser radar camera system using a sphere. Verma et al. [11] manually extracted calibration plate feature points to obtain the required features in two sensor frames in order to obtain external calibration parameters between the camera and the LiDAR. Wang et al. [12] developed a full-size model of a chessboard to achieve the external calibration of LiDAR and panoramic cameras. Xie et al. [13] proposed a pixel and 3D point alignment method based on manually extracting calibration plate feature points to achieve the fusion of LiDAR and camera data. Zhou et al. [14] solved the external calibration problem of cameras and 3D LiDAR sensors using a chessboard. Zhang et al. [15] calibrated the internal parameters of the camera and LiDAR sensor using the chessboard mode and obtained their external parameters.

Owing to the clear planar features and advantages of chessboard patterns, such as high precision, ease of implementation, and good stability, extensive research has been conducted on their use. Deng et al. [16] proposed a correlation joint calibration method using a circular chessboard. Liu et al. [17] proposed an external calibration method between a laser radar system and a camera using a specially designed circular calibration plate. Debattisti et al. [18] used triangular plates, Pereira  et al. [19] used spheres, and Pusztai et al. [20] used boxes as calibration targets for the joint calibration of LiDAR and cameras. The diversity of this shape ensures that the target is easily distinguishable among the sensor data; however, this method has a low calibration accuracy for dynamic calibration objects. Xu et al. [21] proposed a LiDAR camera calibration method based on an improved random sample consensus (RANSAC) algorithm. This method calculates the calibration plate plane and edge parameters based on random sample consistency, determines the positional relationship between the two sensors, and completes joint calibration. Peng et al. [22] proposed a LiDAR camera external parameter calibration algorithm based on semantic information and completed sensor joint calibration based on image registration and perspective-n-point matching sensor semantic information. The advantages of these target-based calibration methods include their high accuracy and usefulness for designing different calibration targets for different application scenarios. However, goal-based methods require special equipment and complex processes. In some cases, there may be errors in object shape recognition, especially at long distances or when the radar point cloud is sparse, leading to difficulties in recognition. For irregular or complex-shaped objects, the performance of this method is poor, and its applicability is limited. Additionally, since precise shape matching is required, these methods consume significant computational resources, particularly in real-time systems.

Targetless methods do not require specific targets during the calibration process. They perform a statistical analysis and modeling of spatial or texture information in the environment to calculate the external parameters between the camera and LiDAR sensors [23]. Schneider et al. [24] constructed a convolutional neural-network model consisting of feature extraction, feature matching, and global regression, which can be calibrated without manually extracting feature point-matching calculations. This was the first study to use deep-learning methods for the online calibration of LiDAR–camera extrinsic parameters. However, the preprocessing steps of this method are cumbersome, and the network structure used is relatively simple, resulting in a weaker ability to extract and match features. Duy et al. [25] proposed an online LiDAR–camera joint calibration method based on the deep-learning prediction of external parameters. By calculating the pixel matching between the point cloud images and color images, a network model was constructed for sensor joint calibration. Lv et al. [26] proposed an online calibration network for LiDAR and cameras, LiDAR-Camera Self-calibration Network (LCCNet), which constructs a loss tensor layer and calculates the correlation between color images generated by point cloud projection and depth images. Yuan et al. [27] proposed a laser radar camera calibration method, a DL-based LiDAR-camera calibration method (RGGNet), based on deep learning by considering Riemannian geometry and using deep generative models to learn implicit tolerance models. The aforementioned deep-learning-based methods, however, have limitations, require considerable training, and have high environmental requirements.

Toth et al. [6] proposed a fully automatic external parameter calibration algorithm for LiDAR cameras based on the surface and contour key points of spherical images to predict the external parameters accurately and complete joint calibration. Bai et al. [7] proposed a joint sensor calibration method based on the linear correspondence between LiDAR and camera. This method solves the parameter matrix based on the correspondence between infinitely distant points in two sensor images. Yu et al. [28] proposed a camera–LiDAR external calibration method based on motion and edge matching. These three methods have the advantages of fast calibration speed, low cost, and strong linearity; however, there are often significant errors in estimating sensor motion, resulting in lower calibration accuracy. In summary, these goalless methods have the advantages of simple calibration, no need for additional calibration equipment, and fast calibration. However, in complex environments, target tracking may be affected by occlusion, noise, or other factors, leading to tracking failure. Additionally, the keypoints in the radar point cloud may be sparse, making matching difficult, especially in low-resolution scenarios. Since continuous tracking and processing of multiple frames of data are required, this method consumes significant computational resources.

An analysis of the current research status reveals that most current calibration algorithms have cumbersome calibration steps, long model training times, and poor flexibility, so they cannot meet the practical needs of online real-time calibration in real-life scenarios. To improve the flexibility, robustness, and calibration accuracy of the calibration algorithm, a deep-learning-based joint calibration method for LiDAR cameras was developed in this study. A neural-network model was constructed and trained. This method does not require any special calibration objects and can automatically capture feature points of objects in natural scenes for matching calculations, solving joint calibration matrix parameters, completing joint calibration of the LiDAR-camera [29], and improving the flexibility of calibration algorithms. To enhance the robustness of the algorithm, a mathematical model was constructed for the joint calibration of LiDAR-cameras. To solve the problem of small training data samples and improve calibration accuracy, a data transfer fusion mechanism was employed.

In summary, the contributions of our work are as follows:

(1) A mathematical model was constructed for joint LiDAR–camera calibration. A detailed analysis of the working principles and data characteristics of cameras and LiDAR, the preprocessing of LiDAR point cloud images into standardized two-dimensional depth images, and the sensor joint calibration process were conducted.
(2) A deep-learning-based LiDAR–camera parameter-solving network model was constructed. The model consists of a feature extraction layer, feature matching layer, and feature aggregation layer. It accurately solves the rotational translation parameter matrix to complete the joint calibration of the spatial positions of two sensors.
(3) A data migration–fusion mechanism was introduced to improve the robustness of the sensor relative position offset and improve the prediction accuracy of the network.

The remainder of this article is structured as follows. In Section 2, the relevant theoretical foundations are presented, including the working principles of LiDAR and cameras. In Section 3, the construction of the mathematical model of LiDAR camera joint calibration and the mechanism of data migration and fusion are described. In Section 4, the proposed deep-learning-based parameter estimation model is introduced. The experimental setup and results are presented in Section 5. Finally, in Section 6, a summary is provided, and future prospects for the research findings are discussed.

## 2. Camera and LiDAR Principle and Related Work

The sensor is the main hardware of the autodrive system and is a tool for obtaining information about the surrounding environment and the vehicle itself. Commonly used sensors include laser mines and visual cameras. In this section, the working principles and imaging geometry of LiDAR sensors and camera sensors are introduced.

### 2.1. Camera Working Principal

**Figure 2:**
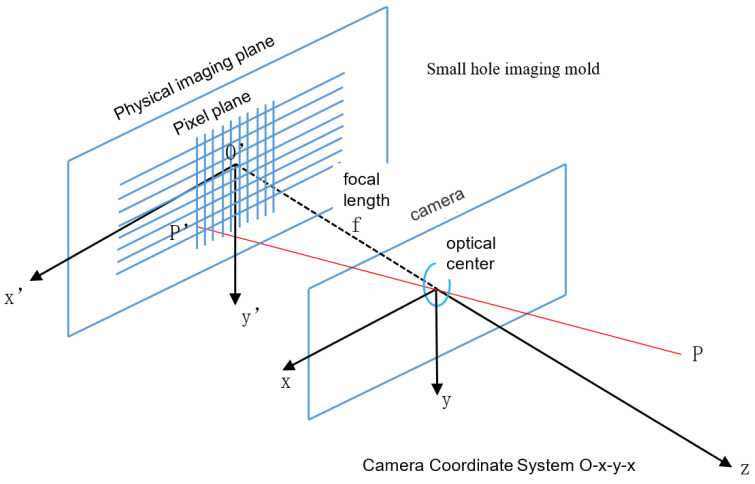
Imaging model.

As a passive sensor, a camera collects three-dimensional environmental information through a photosensitive element, and its imaging process can be simplified into a small-aperture imaging model. Light passes through the optical center of the camera and is projected through transmission to map the three-dimensional environment onto a two-dimensional image plane for imaging (Figure 2). Camera imaging mainly involves four coordinate systems: world coordinate, camera coordinate, image coordinate, and pixel coordinate systems [30].

The world coordinate system is used to annotate the position of the camera in real-world scenes and the position of objects in the environment. It is the reference coordinate that determines the position of the objects on the imaging plane. The camera coordinate system represents the position of an object relative to the camera. When projecting the image, the optical center of the camera is used as the coordinate origin, and the optical axis direction of the camera is set to the *z*-axiswith the *x*-axis and *y*-axis parallel to the image coordinate system.

To clarify the internal relationships between the images, an image coordinate system was established, the image center was set as the origin of the image coordinate system, and the *x*-axis and *y*-axis of the image coordinate system were set as vertical edges parallel to the image plane. A pixel coordinate system was established on the image plane to store an M × A pixel matrix of size N. The pixel coordinate takes the vertex at the top-left corner of the image plane as the coordinate origin, and the pixel coordinate *x*-axis and *y*-axis are parallel to the image coordinate system *x*-axis and *y*-axis.

### 2.2. Camera Imaging Geometry

Before calibrating the LiDAR and camera, it is necessary to calibrate the monocular camera to obtain the parameters for the imaging geometry. By analyzing the camera imaging process, one can understand how points in the three-dimensional world correspond to the camera image. This forms the basis for aligning the LiDAR point cloud with the camera image data.

**Figure 3:**
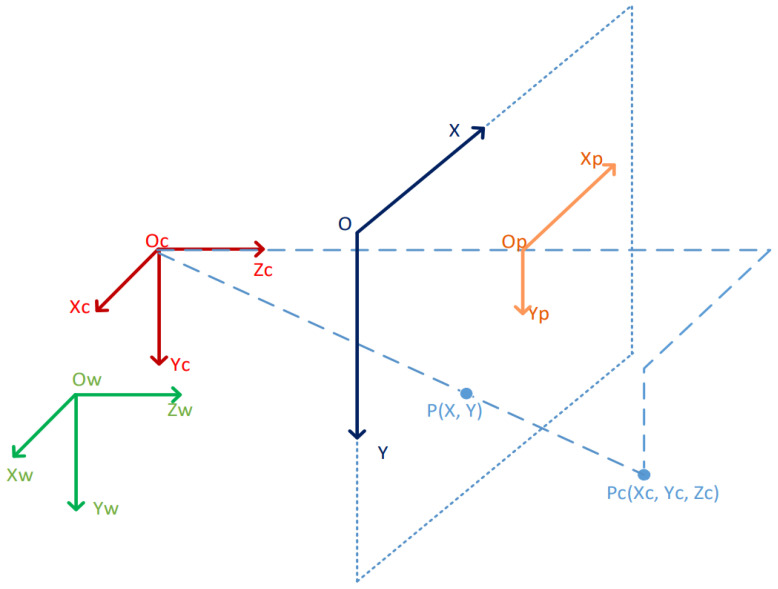
The four coordinate systems in camera imaging geometry.

The camera’s imaging geometry involves four coordinate systems, as shown in Figure 3. These include two three-dimensional coordinate systems: the world coordinate system ($O_{w}$-$X_{w}Y_{w}Z_{w}$) and camera coordinate system ($O_{c}$-$X_{c}Y_{c}Z_{c}$), as well as two two-dimensional coordinate systems: the image coordinate system ($O_{p}$-$X_{p}Y_{p}$) and pixel coordinate system (*O*-XY).

In the world coordinate system, the $X_{w}$ axis represents the horizontal axis of the world, the $Y_{w}$ axis represents the vertical axis of the world, and the $Z_{w}$ axis represents the depth axis of the world. In the camera coordinate system, the $X_{c}$ axis represents the horizontal axis of the camera coordinate system, the $Y_{c}$ axis represents the vertical axis of the camera coordinate system, and the $Z_{c}$ axis represents the depth axis of the camera coordinate system. In the image coordinate system, the $X_{p}$ axis represents the horizontal physical coordinate of the image plane, and the $Y_{p}$ axis represents the vertical physical coordinate of the image plane. In the pixel coordinate system, the *X* axis represents the horizontal pixel position in the image, and the *Y* axis represents the vertical pixel position in the image.

The relationship between the world coordinate system and the camera coordinate system is defined by a rigid-body transformation. Here, $P_{c}$ = ${\left[x_{c},y_{c},z_{c},1\right]}^{T}$ is the homogeneous representation of a point in the camera coordinate system, and $P_{w}$ = ${\left[x_{w},y_{w},z_{w},1\right]}^{T}$ is the homogeneous representation of a point in the world coordinate system.

The relationship between the camera coordinate system and the image coordinate system is defined by a perspective projection, as shown in Equation (1).

(1) $$\begin{array}{c} P_{p}=\left[\begin{array}{ccc} f/z_{c} & 0 & 0\\ 0 & f/z_{c} & 0\\ 0 & 0 & 1 \end{array}\right]P_{c}\; \end{array}$$

where $P_{p}$ = ${\left[x_{p},y_{p},1\right]}^{T}$ is the homogeneous representation of a point in the image coordinate system, $P_{c}$ = ${\left[x_{c},y_{c},z_{c}\right]}^{T}$ is the point in the camera coordinate system, and *f* is the focal length of the camera.

The transformation between the image coordinate system and the pixel coordinate system primarily involves scaling the coordinate system units and offsetting the coordinate origin, as shown in Equation (2).

(2) $$\begin{array}{c} P=\left[\begin{array}{ccc} 1/s_{x} & 0 & u\\ 0 & 1/s_{y} & v\\ 0 & 0 & 1 \end{array}\right]P_{p}\; \end{array}$$

where $s_{x}$ and $s_{y}$ represent the dimensions of the image pixels along the x-axis and y-axis, respectively, *P* = ${\left[x,y,1\right]}^{T}$ denotes the homogeneous representation of a point in the pixel coordinate system, and (u,v) represents the position of the coordinate system origin in the pixel coordinate system.

By combining the transformations of the four coordinate systems, one can obtain the transformation formula from the world coordinate system to the pixel coordinate system, as shown in Equation (3).

(3) $$\begin{array}{c} P=\left[\begin{array}{c} x\\ y\\ 1 \end{array}\right]=\left[\begin{array}{ccc} f/(s_{x}z_{c}) & 0 & u\\ 0 & f/(s_{y}z_{c}) & v\\ 0 & 0 & 1 \end{array}\right]\left[\begin{array}{c} x_{2}\\ y_{2}\\ 1 \end{array}\right]\\ =\left[\begin{array}{cccc} r_{11} & r_{12} & r_{13} & t_{1}\\ r_{21} & r_{22} & r_{23} & t_{2}\\ r_{31} & r_{32} & r_{33} & t_{3}\\ 0 & 0 & 0 & 1 \end{array}\right]\left[\begin{array}{c} x_{w}\\ y_{w}\\ z_{w}\\ 1 \end{array}\right]\; \end{array}$$

where $R=\left[\begin{array}{ccc} r_{11} & r_{12} & r_{13}\\ r_{21} & r_{22} & r_{23}\\ r_{31} & r_{32} & r_{33} \end{array}\right]$ represents the rotation matrix, and *T* = ${\left[t_{1},t_{2},t_{3}\right]}^{T}$ represents the translation vector, which comprises the extrinsic parameters in the camera calibration process, and $f/(s_{x}z_{c})$, $f/(s_{y}z_{c})$, (u,v) represent the intrinsic parameters in the camera calibration.

### 2.3. Working Principle and Data Characteristics of LiDAR

**Figure 4:**
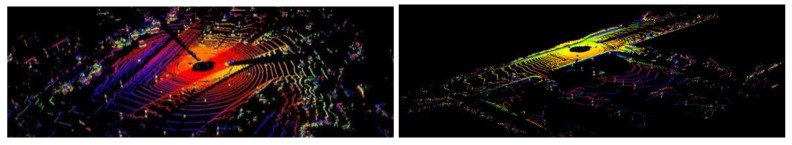
Point clouds of LiDAR.

**Figure 5:**
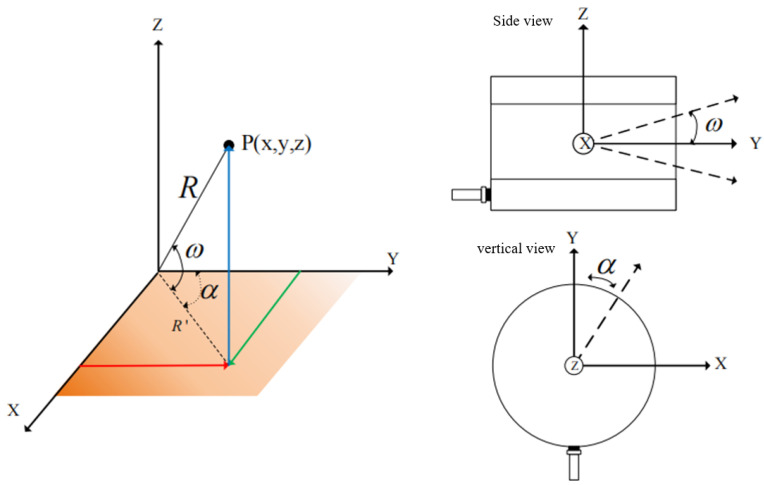
LiDAR point cloud image three-dimensional space coordinate transformation.

LiDAR comprises a laser transmitter, receiving system, and rotating code disk. When LiDAR is in operation, the laser emitter emits a laser beam that detects an object and reflects it back, which is then received by the receiving system. The time difference between the emission and the reception of light is measured, and the distance between the LiDAR and the target is calculated. The rotating encoder drives the laser transmitter to rotate and scan horizontally to obtain a complete point cloud image. Point cloud images are unordered, sparse, and nonuniform cylindrical range views with high accuracy and are not easily affected by changes in environmental light intensity. The resolution is mainly affected by the number of lines in the LiDAR. The higher the number of lines, the higher the resolution of the LiDAR point cloud image. Taking the 64-line LiDAR model Velodyne HDL64 as an example, the horizontal resolution is 0.09°, the vertical resolution is 0.4°, the vertical field-of-view angle is $+2^{\circ }∼-24.8^{\circ }$, the scanning frequency is 5–15 Hz, and each point cloud image collected has approximately 100,000 laser points, as shown in Figure 4. These points are determined by the distance *r*, reflectivity *e*, and azimuth angle θ. The four laser elevation angle ϕ parameters are uniquely determined. To facilitate the further processing of point cloud images and joint calibration with camera color images, the point cloud scattered points are transferred to the LiDAR point cloud coordinate system based on the four parameters and Equation (4) of the range view. The three-dimensional spatial coordinate transformation is shown in Figure 5, and its position in the spatial coordinate system is calculated based on the coordinate axis of the spatial coordinate system and the angle and distance between the scattered points of the point cloud and the coordinate axis [31].

(4) $$\begin{array}{c} P_{l}=\left[\begin{array}{c} x_{l}\\ y_{l}\\ z_{l} \end{array}\right]=\left[\begin{array}{c} rcos\phi cos\theta \\ rcos\phi sin\theta \\ rsin\phi \end{array}\right]\; \end{array}$$

where $p_{l}$ are the points of the LiDAR point clouds, $x_{l}$ is the x-axis coordinates of the points, $y_{l}$ is the *y*-axis coordinates of the points, $z_{l}$ is the z-axis coordinates of the points, ϕ is the upward deviation angle in the vertical direction, and θ is the horizontal upward deviation angle.

**Figure 6:**
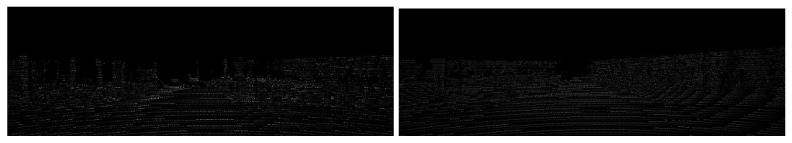
Depth image of point cloud map.

After a point cloud has been converted into a three-dimensional representation in a spatial coordinate system, it is still not possible to perform joint calibration with the point cloud image directly. A point cloud image is a three-dimensional spatial map, whereas a camera image is a two-dimensional plane view. For calibration, the point cloud image is preprocessed and projected onto a two-dimensional plane. The point cloud image is mapped onto the two-dimensional plane according to relevant parameters, such as the size of the corresponding color images collected in the same scene [31], as shown in Figure 6. Projecting a 3D point cloud onto a 2D image plane is a geometric transformation process based on the parameters of a virtual camera. This process mainly involves the intrinsic and extrinsic parameters of the camera, which are used to map each point in the 3D point cloud from the 3D coordinate system to the 2D pixel coordinates of the image. The coordinate mapping is completed in two steps: the first step uses the camera’s extrinsic parameters to transform the 3D points from the world coordinate system to the camera coordinate system, and the second step uses the camera’s intrinsic parameters to project the 3D points in the camera coordinate system onto the 2D image plane.

## 3. Method

In this section, the construction of the mathematical model for the LiDAR–camera joint calibration and the mechanism of data migration and fusion are introduced in detail. A mathematical model of the joint LiDAR–camera calibration is presented, the working principle and data acquisition characteristics of the sensor are analyzed in detail, and the normalized processing of a point cloud image is converted into that of a depth image for further matching and calculation with a color image. In the deep learning process, data migration and fusion mechanisms, such as random rotation, are introduced to improve network prediction accuracy.

### 3.1. Joint Calibration Mathematical Model

**Figure 7:**
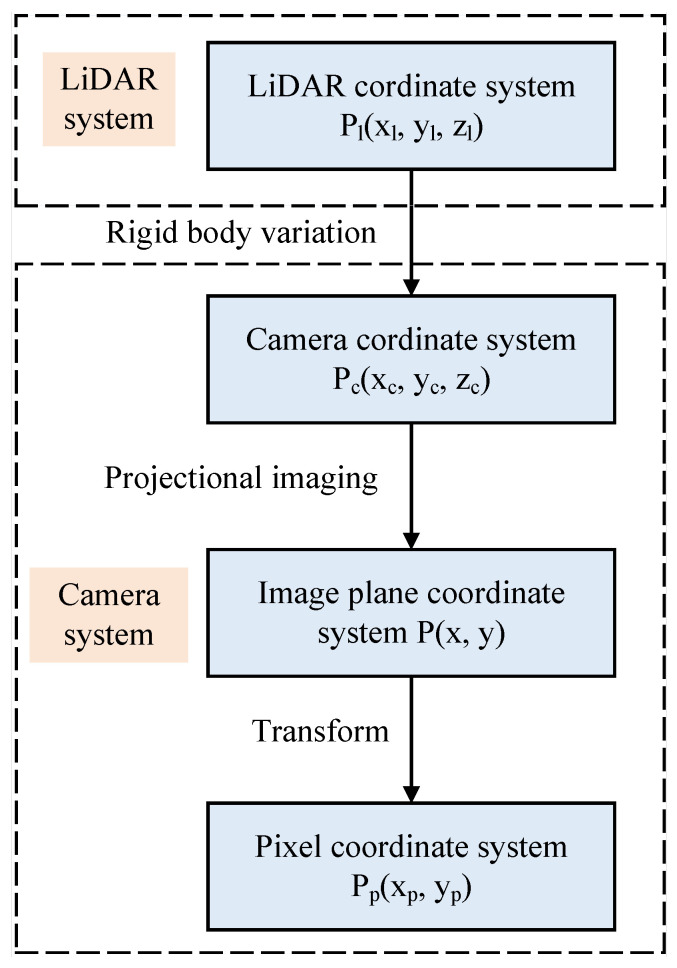
Joint calibration model of LiDAR and camera.

**Figure 8:**
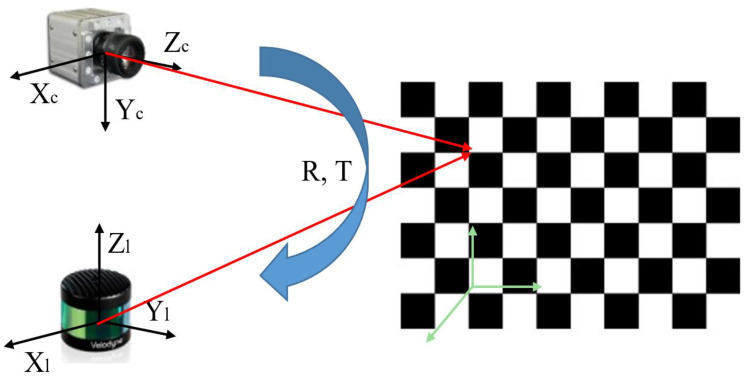
Joint calibration mathematical model.

The joint calibration between the LiDAR and camera sensors was studied, and spatial synchronization and fusion were achieved by jointly calibrating the camera coordinates and LiDAR coordinates. The camera coordinate system was used as the carrier coordinate system to simplify the model and facilitate its description. A mathematical model was constructed to establish the constraint relationship between the camera and LiDAR, and the coordinate conversion process between the two was explained, as shown in Figure 7 and Figure 8. When there is a point *P* in the space scene, the coordinate of the point is $P_{c}$ in the camera coordinate system, and the coordinate of the point cloud image collected by LiDAR is $P_{l}$. As Figure 7 shows, the point cloud scatter set in the LiDAR coordinate system is first mapped to the camera coordinate system through rotation and translation for rigid-body changes, as shown in Equation (5). The rotation matrix and shift matrix parameters must be determined. Current methods include calibration methods based on special calibration objects and methods based on natural scene objects. The coordinates in the coordinate system are mapped to the image plane coordinate system by projection imaging, and the image plane coordinate system is mapped to the pixel coordinate system by geometric transformation.

(5) $$\begin{array}{c} P_{c}={R_{c}}^{l}.P_{l}+{T_{c}}^{l}\; \end{array}$$

where ${R_{c}}^{l}$ is the rotation matrix from the laser radar coordinate system to the camera coordinate system, and ${T_{c}}^{l}$ is the translation matrix from the LiDAR coordinate system to the camera coordinate system.

The rigid-body transformation of rotation and translation in space requires three rotational and three translation parameters. These are represented by a rotation matrix and translation matrix, respectively. The rotation matrix can be determined from the yaw, pitch, and roll angles, as shown in Equation (6).

In Equation (6), φ is the yaw angle, θ is the pitch angle, ψ is the roll angle, sini is the sine angle with the *i*-axis, and cosi is the cosine angle along the *i*-axis (i stands for φ/θ /ψ).

The yaw angle is the angle between the actual heading of the vehicle and the planned heading, the pitch angle is the angle between the vehicle axis and the ground plane, and the roll angle is the angle at which the vehicle rotates around the front and rear axes. The translation matrix can be represented by the translation distance of a point in space on the three axes of the spatial coordinate system, as expressed in Equation (7).

(6) $$\begin{array}{c} {R_{c}}^{l}=\left[\begin{array}{ccc} 1 & 0 & 0\\ 0 & cos\varphi & sin\varphi \\ 0 & -sin\varphi & cos\varphi \end{array}\right]\left[\begin{array}{ccc} cos\theta & 0 & sin\theta \\ 0 & 1 & 0\\ sin\theta & 0 & cos\theta \end{array}\right]\left[\begin{array}{ccc} cos\psi & sin\psi & 0\\ -sin\psi & cos\psi & 0\\ 0 & 0 & 1 \end{array}\right]\\ =\left[\begin{array}{ccc} cos\theta cos\psi & sin\theta sin\psi & -sin\theta \\ sin\varphi sin\theta cos\psi -cos\varphi sin\psi & sin\varphi sin\theta sin\psi +cos\varphi cos\psi & sin\varphi cos\theta \\ cos\varphi sin\theta cos\psi +sin\varphi sin\psi & cos\varphi sin\theta sin\psi -sin\varphi cos\psi & cos\varphi cos\theta \end{array}\right]\; \end{array}$$

(7) $$\begin{array}{c} {T_{c}}^{l}=\left[\begin{array}{c} t_{x}\\ t_{y}\\ t_{z} \end{array}\right]\; \end{array}$$

where $t_{i}$ is the translation distance on the *i*-axis.

### 3.2. Data Migration–Fusion Mechanism

**Figure 9:**
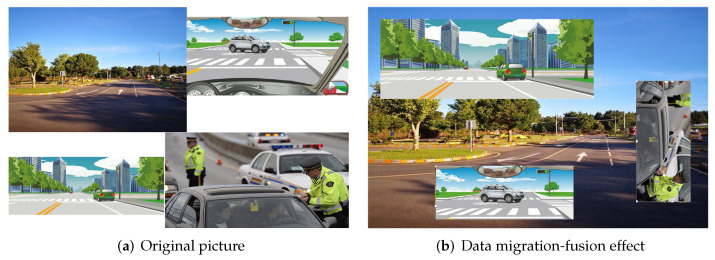
Data migration–fusion mechanism.

To improve the robustness of the neural network to sensor deviation or rotation error, a data migration–fusion mechanism was introduced in the process of network training, and the sample data of the dataset were enriched to enhance it. The principle of the data migration–fusion mechanism is shown in Figure 9. Multiple images are randomly scaled and clipped to generate new images under the same image. Data migration enriches the training-data sample size without modifying the original dataset. Such methods as random rotation, scaling, and clipping enhance the robustness of the network to sensor-offset errors in real scenes. A multi-image mosaic improves the training speed of the neural network and accelerates network convergence. The main advantages of this method are as follows.

(1) Rich dataset: Randomly using multiple images, randomly scaling, and then splicing the random distribution substantially enriches the detection dataset. In particular, random scaling adds many small targets, making the network more robust.
(2) GPU memory reduction: The data of multiple pictures are directly calculated such that the minibatch size need not be large to achieve better results.

The specific implementation steps are shown in Algorithm 1.   
**Algorithm 1:** Data migration–fusion algorithm. **Input**: pictures: Collection of original images; input_shape: specifies the size of the image
 **Output**: image_new_list: a collection of new images enhanced with data
1 image_list=get_image_info(Picture); 2 image_datas, box_data=get_random(image_list, input_shape); 3 new_images=Merge_image(image_datas); 4 image_new_list =Merge_boxes(new_images,box_data); 5 Return image_new_list


First, the image information is obtained (Line 1). After the image is obtained, it is divided according to the preset image size and the existing image (Line 2). The cut picture is rotated and then spliced (Line 3). Finally, the detection box boundary is processed to obtain the final new image collection (Line 4).

## 4. Deep Learning Parameter Solving Model

To solve for the parameters of the constructed joint calibration data model, the parameter-solving model based on deep learning proposed in this study is provided in detail.

### 4.1. LiDAR-Camera Joint Calibration Network Model

The deep-learning-based calibration parameter estimation model presented utilizes a calibration method based on natural-scene objects. The model employs a convolutional neural network to process a large dataset and extract features from objects in natural scenes. Once the network is trained and converges, it can automatically predict the joint calibration extrinsic parameters based on the images captured by the sensors, thus achieving automatic calibration.

**Figure 10:**
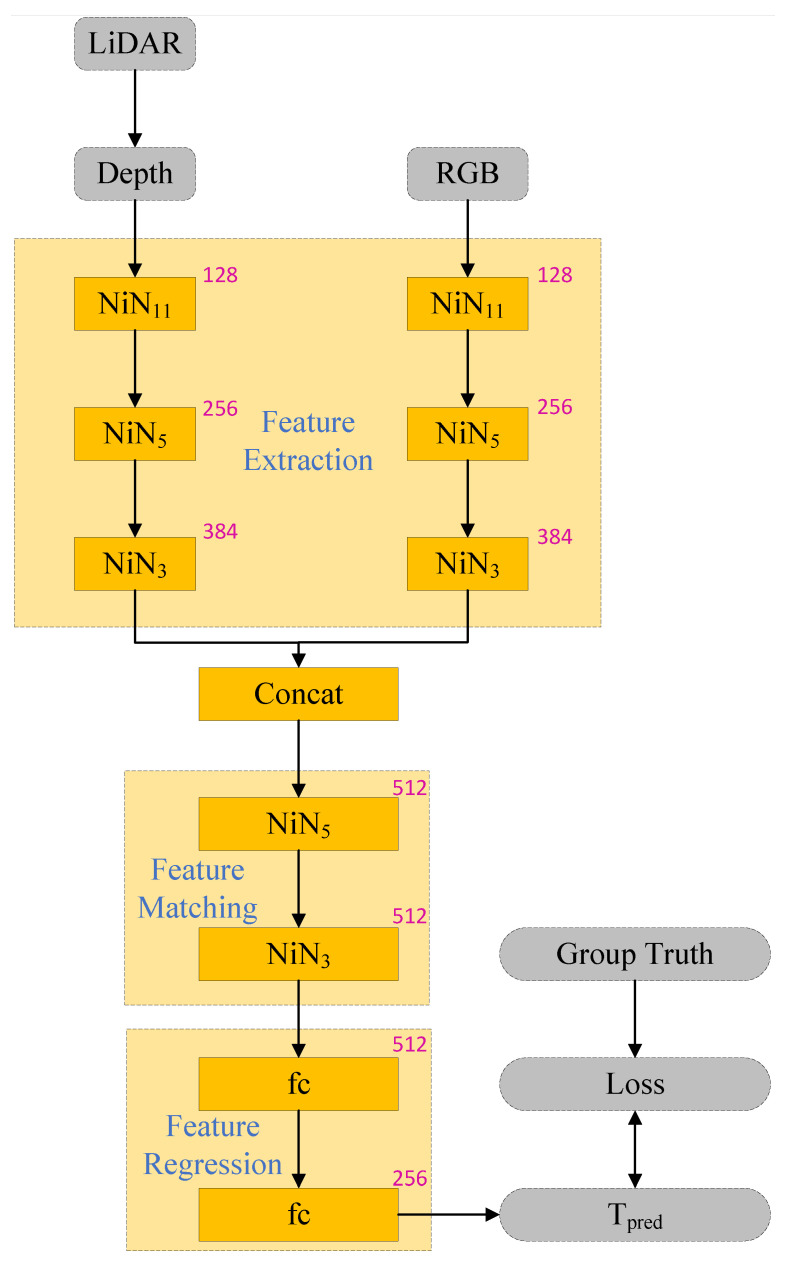
LiDAR-camera joint calibration model.

The constructed calibration parameter estimation model has the structure shown in Figure 10, consisting of feature extraction, feature matching, and feature regression modules. These three modules are combined into a single network, making end-to-end training possible. The input of the network consists of red–green–blue (RGB) images captured by the camera and depth images obtained by projecting the point cloud data collected by LiDAR. The network outputs a parameter matrix $T_{pred}$, which contains three rotation parameters and three translation parameters. During the training process, the deviation between the predicted values $T_{pred}$ and the ground truth labels $T_{gt}$ is computed, and the error is corrected continuously through backpropagation. Once the neural-network training converges, accurate values of the extrinsic parameters can be obtained to complete the joint calibration of multiple sensors.

#### 4.1.1. Feature Extraction Module

The feature extraction module consists of two symmetric branches and employs a modular design to reduce the neural network complexity. It extracts features from both the color image and the depth image. The RGB image branch utilizes network-in-network (NiN) modules and a rectified linear unit (ReLU) activation function, whereas the depth image branch uses the same modules but replaces the ReLU function with a leaky ReLU. We construct a network by arranging several Net-in-Net (NiN) blocks proposed by Lin et al. [32], which consist of a k × k convolution and several 1 × 1 convolutions. The NiN module replaces the fully connected layers with 1 × 1 convolutional layers, enabling spatial information to propagate naturally for feature matching. The feature extraction module fully extracts multimodal data feature maps through convolution and places the extracted features into the feature-matching module for data feature-matching calculation.

#### 4.1.2. Feature Matching Module

The cost volume module is used to calculate the degree of feature matching between the image feature maps, as shown in Equation (8). Matrix multiplication is performed on the feature map vectors extracted from the two feature extraction branches to determine the deviation and average error between the pixel points in the two feature maps. The obtained matching feature vectors are input into the feature regression module to determine the calibration matrix parameter values of the two sensors. The feature-matching module comprises two fully connected layers, and the kernel size *k* of the first convolutional layer of the NiN block is represented by an index. The number of feature channels is 512, as shown in the upper right corner of each layer module in Figure 10.

(8) $$\begin{array}{c} cv\left(P_{RGB},P_{l}\right)=\frac{1}{N}{\left(c\left(X_{RGB}\left(P_{RGB}\right)\right)\right)}^{T}c\left(X_{l}\left(P_{l}\right)\right)\; \end{array}$$

where c(x) is the feature vector of the feature map, *N* is the length of the feature vector c(x), and $cv(x_{1},x_{2})$ is the correlation of the two quantities.

#### 4.1.3. Feature Regression Module

For regression calibration, the global information extracted by the feature-matching module must be collected. To achieve global information fusion, two fully connected layers are superimposed, and then a Euclidean loss function is superimposed. The feature regression module consists of two fully connected layers with 512 and 256 neuronal nodes. The characteristic regression layer outputs the prediction matrix $T_{pred}$.

#### 4.1.4. Regression Loss Function

In the joint calibration model, a smooth $L_{1}$ loss function is used for the translation vector $T_{pred}$. The derivative of the $L_{1}$ loss is not unique at 0, which affects the training convergence. Compared with $L_{1}$ losses, smooth $L_{1}$ losses are smoother because the square function is close to 0. Regarding the rotation loss $L_{q}$, because quaternions are essentially directional information, the Euclidean distance cannot accurately describe the difference between the two quaternions. Therefore, the angular distance is used to represent the difference between the quaternions, as defined in Equation (9).

(9) $$\begin{array}{c} L_{R}=F_{s}\left(T_{gt},T_{pred}\right) \end{array}$$

where $T_{gt}$ is the ground truth of the quaternion, $T_{pred}$ is the prediction, and $F_{s}\left(.\right)$ is the angular distance between the two quaternions. The total regression function $L_{T}$ is a combination of translation and rotation losses, as shown in Equation (10).

(10) $$\begin{array}{c} L_{T}=\alpha_{t}L_{t}+\alpha_{q}L_{R} \end{array}$$

where $L_{t}$ is the smooth $L_{1}$ loss of the translation, and $\alpha_{t}$ and $\alpha_{q}$ indicate their respective loss weights.

#### 4.1.5. Iterative Refinement

To enhance the robustness of the neural network against sensor offsets or rotational errors, a data migration–fusion mechanism is introduced to process the original dataset. Such techniques as translation and random rotation are used to increase the number of data samples and simulate errors caused by sensor position changes, such as rotation and translation. This approach improves the robustness of the neural network to changes in the relative positions of the sensors.

#### 4.1.6. Pseudocode Implementation of Algorithm

Based on the above description of the joint LiDAR–camera calibration, the entire joint calibration algorithm is shown in Algorithm 2.
**Algorithm 2:** Algorithm for solving the joint calibration model of LiDAR and camera.
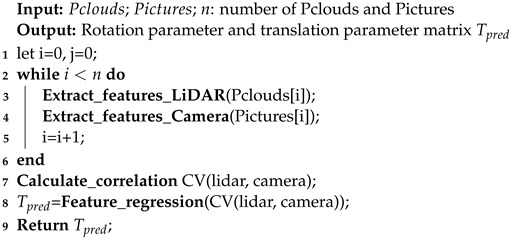


First, the feature vectors of the RGB image captured by the camera and the depth image obtained by projecting the LiDAR point cloud are extracted (Lines 2–6). The matching score between the two feature vectors is calculated using Equation (8) to obtain the matching feature vector (Line 7). The feature-matching vector is input into a feature regression model to compute the rotation parameter matrix and translation parameter matrix $T_{pred}$ (Line 8). Finally, matrix $T_{pred}$ is the output.

## 5. Evaluations

The proposed calibration algorithm was tested and verified. First, the algorithm model was trained. After the neural network converged, the proposed calibration algorithm was compared with others to verify its high performance. To demonstrate the effectiveness of the proposed algorithm further, calibration examples were visualized. Finally, the validity of the data migration and fusion mechanisms was verified.

### 5.1. Settings

#### 5.1.1. Experimental Dataset

The Karlsruhe Institute of Technology and Toyota Technological Institute (KITTI) odometry dataset [33] was used to test and validate the proposed calibration algorithm. The data collection platform for the KITTI dataset was equipped with two grayscale cameras, two color cameras, a Velodyne 64-line LiDAR, and a GPS navigation system. The KITTI odometry dataset consists of 21 data sequences collected in different scenarios and provides calibration parameters for all sensors in different scenarios. The calibration parameters were used for the LiDAR and left-color camera. Data from the 01–20 sequence (39,011 frames) were used as training and validation samples, and data from the 00 sequence (4541 frames) were used as test samples.

#### 5.1.2. Experimental Environment

The experimental environment for this study was a Lenovo ThinkStation workstation and Ubuntu 20.04 operating system with a built-in Nvidia GTX2070Ti graphics card and 32 GB of running memory. The software environment was based on Anaconda3 and included virtual environment drivers, such as cuda10.2, cudnn7.6.8, and gcc4.5.6. The program was run using the Python 3.6 programming language and the Python deep-learning framework, as listed in Table 1.

### 5.2. Effectiveness

To evaluate the proposed method, several experiments were conducted using real sensor data. Because the primary interest was in sensors related to autonomous driving, the focus was on the calibration of the LiDAR–camera settings. First, the effectiveness of the model was validated based on the training speed and different calibration ranges. Subsequently, the progressiveness of this algorithm was verified by comparing it with other advanced algorithms. Finally, the effectiveness of the data migration and fusion mechanism proposed was verified experimentally.

#### 5.2.1. Network Model Training Convergence

To evaluate the translation errors on the *x*-, *y*-, and *z*-axes and the rotation errors around the three axes, the network optimizer used the Adam optimizer function when training the network model. The other parameters are listed in Table 2.

**Figure 11:**
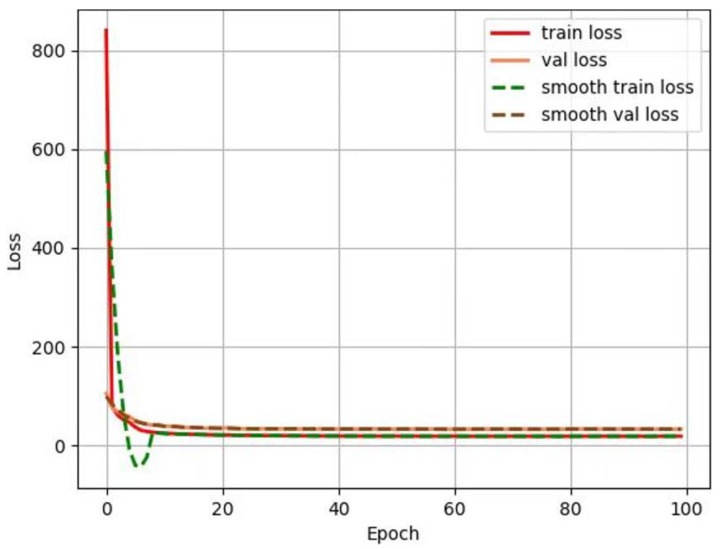
Network model training results.

By constantly adjusting the learning rate and other model parameters, optimal training results were obtained. The “train loss” and “val loss” in Figure 11 measure the fitting and generalization abilities of the model on the training set, respectively. As the figure shows, both loss function curves converge synchronously and quickly in the end, indicating that the model has strong generalization ability and high efficiency.

#### 5.2.2. Different Decalibration Ranges

The network models were trained on different recalibration ranges based on the worst mean absolute error of the network and trained on the next-larger range to achieve higher robustness. The following ranges were determined: [−x,x]/[−y,y] (translation/rotation) x = {1.5 m, 1.0 m, 0.5 m, 0.2 m, 0.1 m} and y = {$20^{\circ }$, $10^{\circ }$, $5^{\circ }$, $2^{\circ }$, $1^{\circ }$}. The final calibration results are listed in Table 3.

Table 3 shows that after multiple range iterations, the calibration error is further reduced, and the error distribution is concentrated on a smaller value. The method achieved a mean square translation error of 0.903 cm with an average translation error of 0.278 cm (x, y, and z are 0.194, 0.297, and 0.342 cm, respectively), an average rotation angle error of 0.134°, and an average angle error of 0.022° (roll, pitch, and yaw = $0.026^{\circ }$, $0.009^{\circ }$, $0.032^{\circ }$).

#### 5.2.3. Average Error and Rotation Error Effect Verification

After neural network convergence, to verify the effectiveness of the algorithm proposed, the calibration algorithm was compared with other calibration algorithms. As shown in Table 4, the proposed approach was compared with previous results [24,25,26] on the KITTI datasets. The uniform mean error range was set to [−1.5 cm, 1.5 cm] and the rotation error range was set to $\left[-20^{\circ },20^{\circ }\right]$.

Schneider [24] constructed a convolutional neural network model consisting of feature extraction, feature matching, and global regression, and calibration could be completed without manual extraction of the feature point matching calculation. The average rotation error and displacement error were ($0.28^{\circ }$, 6 cm) on KITTI, which was much larger than the error value in this study. Calibnet [25] is an end-to-end deep neural network for directly predicting the extrinsic parameters, and its average rotation error and translation error on KITTI were ($0.4^{\circ }$, 4.2 cm), which are larger than those in this study.

LCCnet [26], an online LiDAR–camera calibration network, calculates the correlation between color images and depth images generated by point cloud projection by constructing a loss tensor layer and uses the $L_{1}$-loss function to calculate the errors between the two and reverse propagation to eliminate the errors to complete calibration. It is a relatively advanced algorithm. Compared with LCCnet on the *x*-, *y*-, and *z*-axes, the translation errors of the algorithm are 0.07 cm, 0.09 cm and 0.51 cm smaller, respectively; the average translation error is 0.13 cm smaller; the rotation angle, pitch angle, and yaw angle errors are $0.28^{\circ }$, $0.41^{\circ }$ and $0.07^{\circ }$ smaller; and the average deflection error is $0.257^{\circ }$ smaller.

Through the above comparative experiments with the three advanced algorithms, the calibration error in this study is significantly lower than that of the other advanced algorithms regardless of translation error or rotation error, which verifies the good calibration performance of the proposed algorithm.

#### 5.2.4. Sample Calibration Case Experiment

**Figure 12:**
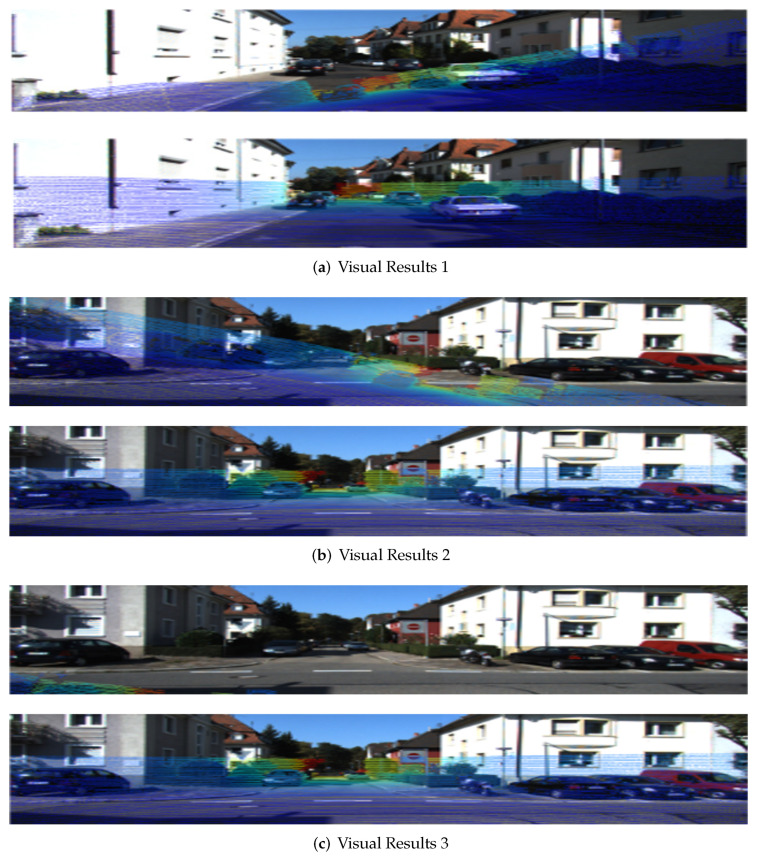
Joint calibration visualization results.

To demonstrate the effectiveness of the proposed calibration algorithm further, some calibration sample cases were visualized (Figure 12).

After training on the rich dataset generated by the data migration–fusion mechanism, the calibration model automatically identifies the environmental features of the changing scene. As Figure 12a shows, the calibration algorithm proposed can fully capture the characteristics of environmental elements in real scenes through deep-learning models, accurately predict the calibration parameters between the LiDAR and camera sensors, and maintain a one-to-one correspondence between the calibrated point cloud scatter points and color image pixels even without special calibration objects in the scene.

The data migration–fusion mechanism was introduced to process the original dataset, and the translation and random rotation methods were used to increase the data sample size to simulate the errors caused by the relative position of the sensor, such as rotation and shift, and to improve the robustness of the neural network to changes in the relative position of the sensor. As Figure 12b shows, the calibration algorithm proposed can predict the correct calibration parameter values and calibrate the two sensors even when the initial LiDAR–camera position differs. It has been proven that the calibration algorithm proposed has good robustness against the initial position deviation of the sensor and can accurately recalibrate the sensor carried by the AGV when it deviates.

In the joint calibration model, the cost volume module is used to calculate the matching degree between the feature maps of the image, and the error is constantly corrected by backpropagation to improve the matching degree between the feature maps. As Figure 12c shows, the calibration algorithm proposed accurately predicts calibration parameters even when the initial positions of the two sensors deviate significantly and only a few scattered points of the LiDAR can be captured.

By using the proposed algorithm to calibrate the samples in the above scenarios, the calibration parameters can be accurately predicted, verifying the high performance of the proposed algorithm model.

#### 5.2.5. Verification of Data Migration-Fusion Mechanism

To verify the effectiveness of the data migration and fusion mechanism proposed, the training models without and with the addition of this mechanism were tested. The test data are listed in Table 5. Comparative experiments showed that adding a data migration and fusion mechanism to the model results in a lower calibration error rate than not adding a data migration and fusion mechanism.

## 6. Conclusions

A deep-learning-based LiDAR–camera joint calibration method was proposed. Unlike most previous solutions, the proposed method does not require other calibration aids. The process involves two stages. First, to simplify the model and describe the camera coordinate system, a mathematical model of LiDAR–camera joint calibration was established. Subsequently, a network model based on deep learning was constructed to capture and match the target features collected by different sensors, and the calibration parameters were calculated. To enhance the robustness of the network to sensor deviations, data migration fusion technologies were introduced, such as rotation and translation. Through simulations and experiments, the performance of the system was verified, and it was proven that it could achieve accurate calibration without other calibration aids. Compared with other nontarget calibration methods, this method has a higher calibration accuracy.

However, several questions remain to be addressed in future research.

(1) Although this study achieved calibration in various scenarios, the presence of noise and changes in lighting conditions can still lead to feature loss. In the future, the deep-learning network model should be optimized to improve its generalization ability.
(2) For the proposed joint calibration algorithm, experimental tests were conducted based on an open-source dataset in a laboratory environment, but the program was not embedded in an AGV for on-site testing in the factory. In future studies, it should be embedded in AGVs in the field. The stability, real-time performance, and detection accuracy of the algorithm should be tested.
(3) Calibration between the camera and the LiDAR sensor is the basis for a higher level of fusion between the two sensors. However, the fusion of the feature level and decision level requires additional algorithms. Plans exist to take the next step using multisensor fusion technology.
(4) Existing calibration methods perform poorly in long-distance scenarios or when the LiDAR point cloud is sparse. Future research will focus on improving calibration accuracy in these situations, potentially by utilizing more complex feature extraction methods or multi-frame data fusion to address these challenges.
(5) Given the differences in data characteristics between different types of sensors (e.g., point clouds and images), future research may explore cross-domain calibration methods, enabling effective data fusion from different sensors in highly heterogeneous environments.

## Figures and Tables

**Table 1:** The experiment environment of this chapter.

Hardware Platform		Software Platform	
CPU	i7 @2.5 GHz	Operating system	Ubuntu
GPU	GTX2070Ti	Deep learning space	Pytorch
RAM	32G	Programming language	Python
Video memory		32G	

**Table 2:** Network training parameter setting.

Network Training Parameter Setting			
Batch _size	32	Epochs size	100
Optimizer	Adam	Weight decay	0.0001
Learning rate	0.003	Iterations	120

**Table 3:** The results of the multi-range network iteration.

Multi-Range	Indicators	Translation Error (cm)				Rotation Error (°)			
		$E_{t}$	X	Y	Z	$E_{R}$	Roll	Pitch	Yaw
After $1^{\circ }$/0.1 m network	Mean	0.903	0.194	0.297	0.342	0.134	0.026	0.009	0.032
	Median	0.721	0.211	0.322	0.292	0.102	0.021	0.012	0.021
	Std.	0.974	0.043	0.193	0.191	0.303	0.011	0.029	0.029
After $2^{\circ }$/0.2 m network	Mean	1.724	0.427	0.431	0.371	0.213	0.120	0.054	0.076
	Median	1.329	0.314	0.348	0.287	0.186	0.084	0.042	0.039
	Std.	1.642	0.321	0.394	0.194	0.377	0.097	0.054	0.082
After $5^{\circ }$/0.5 m network	Mean	2.378	0.986	1.829	0.896	0.374	0.236	0.211	0.214
	Median	2.211	0.913	1.714	0.974	0.246	0.176	0.184	0.119
	Std.	1.812	0.512	0.6220	0.324	0.537	0.214	0.413	0.243
After $10^{\circ }$/1.0 m network	Mean	3.987	1.378	2.231	1.238	0.469	0.293	0.314	0.324
	Median	3.724	1.394	2.574	1.144	0.314	0.189	0.209	0.213
	Std.	2.471	0.714	0.987	0.589	0.674	0.513	0.577	0.398
After $20^{\circ }$/1.5 m network	Mean	5.782	2.410	3.047	3.228	0.631	0.534	0.582	0.603
	Median	5.210	2.340	3.141	2.874	0.811	0.319	0.412	0.372
	Std.	3.971	0.994	1.682	1.019	1.144	0.919	0.891	0.602

**Table 4:** Comparison results of calibration algorithms with other advanced calibration algorithms on the KITTI–odometry dataset.

Method	Error Range	Translation Error (cm)				Rotation Error (°)			
		Mean	X	Y	Z	Mean	Roll	Pitch	Yaw
Regnet [24]	[−1.5 m, 1.5 m]/[$-20^{\circ }$, $20^{\circ }$]	6	7	7	4	0.28	0.24	0.25	0.36
Calibnet [25]	[−1.5 m, 1.5 m]/[$-20^{\circ }$, $20^{\circ }$]	4.2	4	1.5	7.2	0.4	0.17	0.9	0.14
LCCnet [26]	[−1.5 m, 1.5 m]/[$-20^{\circ }$, $20^{\circ }$]	0.49	0.32	0.35	0.8	0.26	0.3	0.42	0.08
Ours	[−1.5 m, 1.5 m]/[$-20^{\circ }$, $20^{\circ }$]	0.26	0.25	0.26	0.29	0.013	0.02	0.01	0.01

**Table 5:** Data migration–fusion mechanism.

Method	Error Range	Translation Error (cm)				Rotation Error (°)			
		Mean	X	Y	Z	Mean	Roll	Pitch	Yaw
Ours	[−1.5 m, 1.5 m]/[$-20^{\circ }$, $20^{\circ }$]	0.26	0.25	0.26	0.29	0.013	0.02	0.01	0.01
Not added	[−1.5 m, 1.5 m]/[$-20^{\circ }$, $20^{\circ }$]	0.30	0.26	0.28	0.29	0.016	0.03	0.02	0.01

## Data Availability

Data are contained with in the article.

## References

[1] Yeong D.J., Velasco-Hernandez G., Barry J., Walsh J. (2021). Sensor and sensor fusion technology in autonomous vehicles: A review. Sensors.

[2] Song W., Zou S., Tian Y., Sun S., Qiu L. (2018). A CPU-GPU hybrid system of environment perception and 3D terrain reconstruction for unmanned ground vehicle. J. Inf. Process. Syst..

[3] Caltagirone L., Bellone M., Svensson L., Wahde M. (2019). LIDAR-camera fusion for road detection using fully convolutional neural networks. Robot. Auton. Syst..

[4] Lee J.S., Park T.H. (2020). Fast road detection by cnn-based camera–lidar fusion and spherical coordinate transformation. IEEE Trans. Intell. Transp. Syst..

[5] Nie J., Yan J., Yin H., Ren L., Meng Q. (2021). A Multimodality Fusion Deep Neural Network and Safety Test Strategy for Intelligent Vehicles. IEEE Trans. Intell. Veh..

[6] Tóth T., Pusztai Z., Hajder L. Automatic LiDAR-camera calibration of extrinsic parameters using a spherical target. Proceedings of the 2020 IEEE International Conference on Robotics and Automation (ICRA).

[7] Bai Z., Jiang G., Xu A. (2020). LiDAR-Camera Calibration Using Line Correspondences. Sensors.

[8] Sengupta A., Ye Y., Wang R., Liu C., Roy K. (2019). Going deeper in spiking neural networks: VGG and residual architectures. Front. Neurosci..

[9] Geiger A., Moosmann F., Car O., Schuster B. Automatic camera and range sensor calibration using a single shot. Proceedings of the IEEE International Conference on Robotics & Automation.

[10] Guo C.X., Roumeliotis S.I. An analytical least-squares solution to the line scan LIDAR-camera extrinsic calibration problem. Proceedings of the 2013 IEEE International Conference on Robotics and Automation.

[11] Verma S., Berrio J.S., Worrall S., Nebot E. Automatic extrinsic calibration between a camera and a 3D Lidar using 3D point and plane correspondences. Proceedings of the 2019 IEEE Intelligent Transportation Systems Conference (ITSC).

[12] Wang W., Sakurada K., Kawaguchi N. (2017). Reflectance intensity assisted automatic and accurate extrinsic calibration of 3d lidar and panoramic camera using a printed chessboard. Remote Sens..

[13] Xie S., Yang D., Jiang K., Zhong Y. (2019). Pixels and 3-D Points Alignment Method for the Fusion of Camera and LiDAR Data. IEEE Trans. Instrum. Meas..

[14] Zhou L., Li Z., Kaess M. Automatic extrinsic calibration of a camera and a 3D lidar using line and plane correspondences. Proceedings of the 2018 IEEE/RSJ International Conference on Intelligent Robots and Systems (IROS).

[15] Zhang Q., Pless R. Extrinsic calibration of a camera and laser range finder (improves camera calibration). Proceedings of the 2004 IEEE/RSJ International Conference on Intelligent Robots and Systems (IROS) (IEEE Cat. No. 04CH37566).

[16] Deng Z., Xiong L., Yin D., Shan F. (2020). Joint Calibration of Dual Lidars and Camera Using a Circular Chessboard.

[17] Liu H., Xu Q., Huang Y., Ding Y., Xiao J. (2023). A Method for Synchronous Automated Extrinsic Calibration of LiDAR and Cameras Based on a Circular Calibration Board. IEEE Sens. J..

[18] Debattisti S., Mazzei L., Panciroli M. Automated extrinsic laser and camera inter-calibration using triangular targets. Proceedings of the 2013 IEEE Intelligent Vehicles Symposium (IV).

[19] Pereira M., Silva D., Santos V., Dias P. (2016). Self calibration of multiple LIDARs and cameras on autonomous vehicles. Robot. Auton. Syst..

[20] Pusztai Z., Hajder L. Accurate calibration of LiDAR-camera systems using ordinary boxes. Proceedings of the IEEE International Conference on Computer Vision Workshops.

[21] Xu X., Zhang L., Yang J., Liu C., Xiong Y., Luo M., Tan Z., Liu B. (2021). LiDAR–camera calibration method based on ranging statistical characteristics and improved RANSAC algorithm. Robot. Auton. Syst..

[22] Jiang P., Osteen P., Saripalli S. Semcal: Semantic lidar-camera calibration using neural mutual information estimator. Proceedings of the 2021 IEEE International Conference on Multisensor Fusion and Integration for Intelligent Systems (MFI).

[23] Wendt A. (2007). A concept for feature based data registration by simultaneous consideration of laser scanner data and photogrammetric images. ISPRS J. Photogramm. Remote Sens..

[24] Schneider N., Piewak F., Stiller C., Franke U. RegNet: Multimodal sensor registration using deep neural networks. Proceedings of the 2017 IEEE Intelligent Vehicles Symposium (IV).

[25] Duy A.N., Yoo M. Calibration-Net: LiDAR and camera auto-calibration using cost volume and convolutional neural network. Proceedings of the 2022 International Conference on Artificial Intelligence in Information and Communication (ICAIIC).

[26] Lv X., Wang B., Dou Z., Ye D., Wang S. LCCNet: LiDAR and camera self-calibration using cost volume network. Proceedings of the IEEE/CVF Conference on Computer Vision and Pattern Recognition.

[27] Yuan K., Guo Z., Wang Z.J. (2020). RGGNet: Tolerance Aware LiDAR-Camera Online Calibration With Geometric Deep Learning and Generative Model. IEEE Robot. Autom. Lett..

[28] Yu Y., Fan S., Li L., Wang T., Li L. (2023). Automatic Targetless Monocular Camera and LiDAR External Parameter Calibration Method for Mobile Robots. Remote Sens..

[29] Huang J.K., Grizzle J.W. (2020). Improvements to target-based 3D LiDAR to camera calibration. IEEE Access.

[30] Nakano T., Sakai M., Torikai K., Suzuki Y., Takeda S., Noda S.e., Yamaguchi M., Nagao Y., Kikuchi M., Odaka H. (2020). Imaging of 99mTc-DMSA and 18F-FDG in humans using a Si/CdTe Compton camera. Phys. Med. Biol..

[31] Zhang K., Ren W., Luo W., Lai W.S., Stenger B., Yang M.H., Li H. (2022). Deep image deblurring: A survey. Int. J. Comput. Vis..

[32] Lin M. (2013). Network in network. arXiv.

[33] Geiger A., Lenz P., Stiller C., Urtasun R. (2013). Vision meets robotics: The KITTI dataset. Int. J. Robot. Res..

